# Primary idiopathic silent chylopericardium

**DOI:** 10.1186/1749-8090-8-28

**Published:** 2013-02-19

**Authors:** Jong Bum Kwon, Si Young Choi, Chi Kyung Kim, Chan Beom Park

**Affiliations:** 1 Department of Thoracic and Cardiovascular Surgery, Daejeon St. Mary’s Hospital, The Catholic University of Korea, Seoul, Republic of Korea; 2 Department of Thoracic and Cardiovascular Surgery, Uijeongbu St. Mary’s Hospital, The Catholic University of Korea, Seoul, Republic of Korea; 3 Department of Thoracic and Cardiovascular Surgery, St. Paul’s Hospital, The Catholic University of Korea, Seoul, Republic of Korea

**Keywords:** Chylopericardium, Pericardial effusion, Pericardial window

## Abstract

Chylopericardium usually occurs secondary to trauma, cardiothoracic surgery, radiation therapy, or neoplasm of the mediastinum. Idiopathic chylopericardium is extremely rare. We report a case of primary chylopericardium in a 79-year-old male patient. Although pericardial window and thoracic duct ligation are the treatment of choice, the patient has been doing well for six months since video-assisted thoracoscopic pericardial window.

## Background

Chylopericardium is a rare and benign disease entity in which chylous fluid accumulates in the pericardial cavity. Chylopericardium developed secondary to iatrogenic after cardiac surgery, trauma, or malignant tumors, including mediastinal tumors. However, primary idiopathic chylopericardium is rarely reported [1,2].

## Case presentation

**Figure 1:**
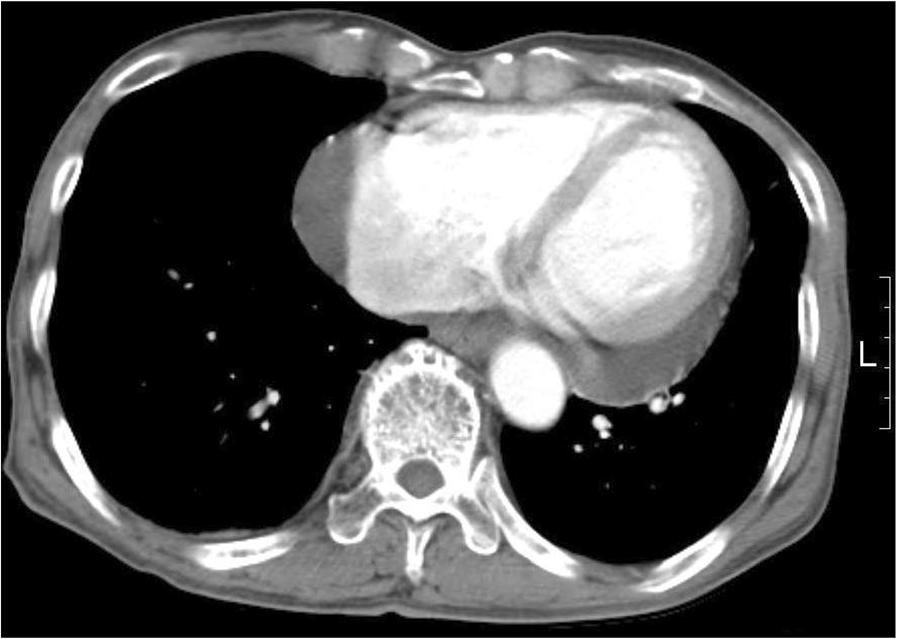
Preoperative computed tomography scan revealed pericardial effusion.

A 79-year-old male was admitted for the management of pericardial effusion. He denied a history of trauma, infection, radiation, mediastinal neoplasm, or cardiothoracic surgery and had a history of adenocarcinoma in the rectum 1 year earlier. The rectal cancer was treated with colonoscopic polyp removal. Pericardial effusion was incidentally found in an abdominal computed tomography (CT) during the evaluation of rectal cancer (Figure 1). Echocardiography showed pericardial effusion (maximum 10 mm depth on diastolic phase) without cardiac tamponade physiology, and an ejection fraction was 70%. Despite medical treatment for 1 year, pericardial effusion was still noted in follow-up echocardiography. He was referred to our department for further evaluation and treatment for pericardial effusion.

Chest x-ray showed an enlarged cardiac shadow. The patient was asymptomatic, his vital signs were stable, and his body temperature was normal. Laboratory findings did not show any signs of inflammation (white blood cell count of 3900/L, erythrocyte sedimentation rate of 3 mm/hr, high sensitivity C-reactive protein concentration of 0.15 mg/dl).

**Figure 2:**
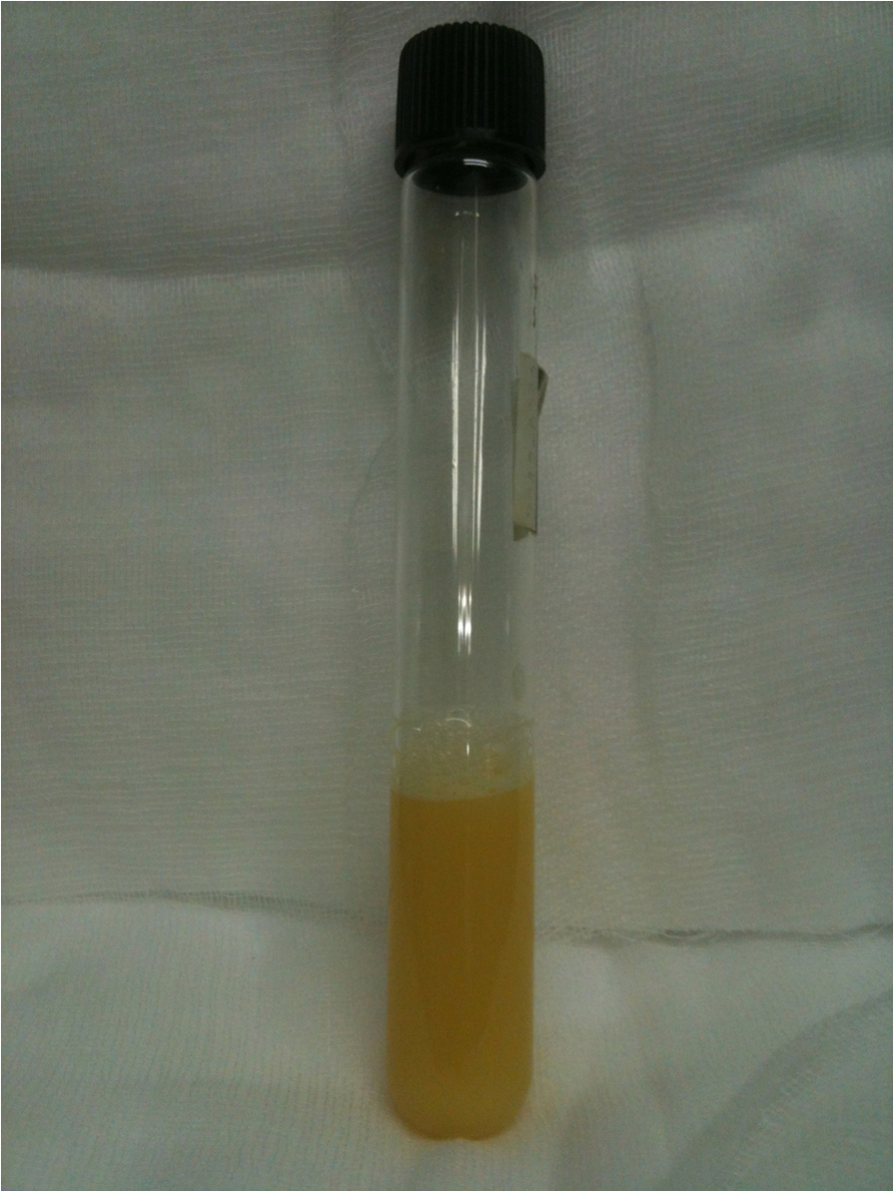
Pericardial fluid, showing a turbid and yellowish appearance.

The patient elected to undergo video-assisted thoracoscopic pericardial window for the evaluation and management of pericardial effusion. At the time of the operation, we thought that tuberculosis was the most probable diagnosis. The patient was placed in right down decubitus position. 10.5 mm port was inserted into the 5^th^ intercostals space midaxillary line and another two 5.5 mm ports were inserted into one inch ahead of the 4^th^ and 6^th^ intercostals space anterior axillary line. The pericardium was grasped and a pericardial window was created. On opening the pericardium, a yellowish, turbid fluid was extracted (Figure 2). Pericardial fluid examination revealed triglyceride 478 mg/dl, total cholesterol 158 mg/dl, and lymphocytes 98%.

Cytologic examination of the pericardial fluid revealed a lymphocyte-dominant cell count (98%) and no evidence of malignant cells. Bacterial cultures were negative, and tuberculosis was excluded. Pathologic examination of the pericardium demonstrated no evidence of malignancy or tuberculosis. A chest CT scan taken after pericardial window did not reveal mediastinal neoplasm, mediastinal lymphadenopathy or other abnormalities that might have obstructed the thoracic duct.

The patient started a normal diet on a routine schedule. Despite the normal diet after operation, the drainage decreased to below 100 ml per day on the day after operation. Although we considered a lymphangiogram, it was not available in our hospital, and the chylous effusion was discontinued. The chest tube was removed on the 5^th^ operative day. Thoracic duct ligation was considered, but the patient did not desire further operations. The postoperative course was uneventful. The patient was followed up 6 months after operation without recurrence of pericardial effusion.

## Discussion

Chylopericardium is a rare cause of pericardial effusion. It sually occurs secondary to trauma, cardiothoracic surgery, radiation therapy, or neoplasm of the mediastinum [3]. Primary idiopathic chylopericardium is extremely rare; recent case reports identified the lymphatic leak and communication with the pericardial sac, which was visualized by lymphangiography, combined lymphangiography and CT, or intraoperative thoracic ductogram [1,2]. While the exact pathophysiology of primary chylopericardium has not been established, the reflux of chylous fluid into the pericardial space has been suggested as an etiology [4]. Damage to the thoracic duct valves and the communication of the thoracic duct to the pericardial lymphatics or abnomally elevated pressure in the thoracic duct could cause chylous fluid reflux.

Chylous fluid is characterized by a milky yellowish appearance, triglycerides >500 mg/dL, a cholesterol/triglyceride ratio < 1.0, a lymphocyte dominant fluid cell count, and negative fluid cultures [3].

Clinical manifestations may vary from asymptomatic, as in our case, to signs of cardiac tamponade. The most common symptoms are dyspnea and fatigue, and chest pain and cough can also be present. While most patients are asymptomatic, conservative treatment is associated with a high recurrence rate (60%) [4] and may lead to pericarditis or cardiac tamponade; therefore, surgical intervention should be considered.

Pericardial effusion is usually diagnosed by chest x-ray, echocardiography or chest CT. Although chylopericardium may be diagnosed by noninvasive imaging using 99 mTc, labeled RBC and the oral administration of 131I-triolein [5], the nature of the effusion is revealed by pericardiocentesis or pericardial window. When the diagnosis of chylopericardium has been established, exclusion of history of trauma and thoracic surgery, chest CT to investigate a secondary cause of chylopericardium is needed. If no other cause of chylopericardium can be found, primary idiopathic chylopericardium is diagnosed. Conventional lymphangiography or CT combined with lymphangiography may reveal abnormalities of the thoracic duct and in some cases demonstrates the presence of communications between the pericardial sac and the lymphatic system [1,6].

Although various management options, including diet therapy with medium-chain triglycerides, pericardiocentesis, pericardial window, and ligation of the thoracic duct have been suggested, the optimal treatment of chylopericardium is unclear. Conservative treatment of idiopathic chylopericardium is usually not satisfactory and a failure rate of 57-60% has been reported [3,4]. Surgical treatment should be considered, even in asymptomatic patients, to avoid subsequent progression to cardiac tamponade or constrictive pericarditis. According to a review by Akamatsu et al [4], the most commonly used surgical method is ligation and resection of the thoracic duct with establishment of pericardial window (41 patients, 52%), pericardial window alone (21 patients, 27%), and ligation and resection of the thoracic duct (7 patients, 9%).

The reaccumulation of chylous fluid in the pericardial space after operation is a major concern following surgical treatment. Thoracic duct ligation and pericardial window are the most effective procedures to prevent recurrence. Pericardial window alone is simple but carries a high incidence of recurrence [2-4] because it does not close the communication between thoracic duct and pericardial sac. Therefore, careful follow up is required, and thoracic duct ligation should be performed if chylopericardium recurs.

Although thoracic duct ligation was not performed in this case, our patient did not show any reaccumulation of chylous pericardial effusion after pericardial window. We assumed that the longstanding chylopericardium and balanced pressure between the pericardial space and thoracic duct eliminated the communication such that the patient remained asymptomatic.

## Conclusion

Although chylopericardium is an uncommon cause of pericardial effusion, clinicians should keep in mind the possibility of chylous effusion. VATS pericardial window is a safe and effective alternative treatment option, although it is associated with a risk of recurrence. In case of reaccumulation, surgical ligation of the thoracic duct should be considered.

## Consent

Written informed consent was obtained from the patient for publication of this Case report and any accompanying images. A copy of the written consent is available for review by the Editor-in-Chief of this journal.

## Abbreviations

CT: Computed tomography.

## Competing interests

The authors declare that they have no competing interests.

## Authors’ contribution

SYC contributed to writing the manuscript. CB Park was responsible for the integrity of the work and edited manuscript. All authors read and approved the final manuscript.
